# Endoscopic submucosal dissection of a large non-pedunculated colorectal polyp using Amber-Red Color Imaging

**DOI:** 10.1055/a-2673-9192

**Published:** 2025-08-27

**Authors:** Luca Brandaleone, Antonio Capogreco, Roberto De Sire, Cesare Hassan, Alessandro Repici, Roberta Maselli

**Affiliations:** 1 551905 Gastroenterology, Endoscopy Unit, IRCCS Humanitas Research Hospital, Rozzano, Milan, Italy; 2 9268 Department of Biomedical Sciences, Humanitas University, Pieve Emanuele, Milan, Italy; 3 9307 Gastroenterology, IBD Unit, Department of Clinical Medicine and Surgery, Università degli Studi di Napoli Federico II, Napoli, Campania, Italy

Amber-Red Color Imaging (ACI), part of the Fujifilm new 800 series platform, is an advanced image-enhancement technology for therapeutic endoscopy. It improves mucosal and vascular contrast during dissection, enabling accurate recognition of tissue planes, clear delineation of lesion margins, and reliable identification of submucosal vessels 1 2 .

We present the case of a 61-year-old man who underwent underwater endoscopic submucosal dissection (U-ESD) for a 40-mm granular mixed-type laterally spreading tumor (LST-GM) located at the recto-sigmoid junction. The lesion showed no endoscopic signs of deep submucosal invasion. MRI confirmed confinement of the tumor with no nodal involvement.

Underwater technique was employed to expand the submucosal space and optimize visualization 3 . Additionally, as saline immersion alters the behavior of electrosurgical currents, it enhances coagulation, improves energy delivery, and reduces the cutting effect 4 . Recently, a novel vessel presealing technique for submucosal third-space endoscopy was introduced, demonstrating high efficacy and cost-effectiveness, and minimizing the need for coagulation forceps 5 . Underwater immersion, vessel identification, and precoagulation were systematically performed. These combined strategies facilitate broader vessel sealing and safer dissection and reduce the risk of intraprocedural bleeding ( Video 1 ).

Granular mixed type lateral spreading tumor (LST-GM) located at the recto-sigmoid junction with visualization of submucosal layer and its vessels using high definition white-light (HD-WL) and ACI, undergoing underwater vessel presealing and therapeutic hemostasis using ACI for vascular enhancement.Video 1

Submucosal bleeding occurred despite prophylactic sealing and was treated successfully using the presealing method (SwiftCOAG E3.0, Erbe VIO3; Erbe Elektromedizin GmbH, Tübingen, Germany).

En bloc resection was achieved, and the mucosal defect was closed using metallic clips. Histopathology confirmed early G2 adenocarcinoma, R0, with lymphovascular invasion (LVI+) and superficial submucosal invasion (pT1 sm2). The patient was subsequently referred to the Colorectal Surgery Unit.

This case highlights the synergistic benefit of combining underwater ESD, ACI, and a novel gastroscope to achieve safer and more precise resection of challenging colorectal lesions. ACI may represent a transformative tool in third-space endoscopy, offering the potential to accelerate procedures and reduce the overall incidence of adverse events.

Endoscopy_UCTN_Code_TTT_1AQ_2AD_3AD

## Notes

Endoscopy E-Videos https://eref.thieme.de/e-videos
*E-Videos* is an open access online section of the journal *Endoscopy* , reporting on interesting cases and new techniques in gastroenterological endoscopy. All papers include a high-quality video and are published with a Creative Commons CC-BY license. Endoscopy E-Videos qualify for HINARI discounts and waivers and eligibility is automatically checked during the submission process. We grant 100% waivers to articles whose corresponding authors are based in Group A countries and 50% waivers to those who are based in Group B countries as classified by Research4Life (see: https://www.research4life.org/access/eligibility/ ). This section has its own submission website at https://mc.manuscriptcentral.com/e-videos .
